# Colonoscopic Splenic Injury: A Simplified Radiologic Approach

**DOI:** 10.1155/2016/2615453

**Published:** 2016-12-18

**Authors:** Tara Chen, Qiu Tong, Alexander Kurchin

**Affiliations:** Department of Surgery, Rochester General Hospital, Rochester, NY, USA

## Abstract

Colonoscopy is a commonly performed procedure for diagnosis and treatment of large bowel diseases. Recognized complications include bleeding and perforation. Splenic injury during colonoscopy is a rare complication. We report a case of a 73-year-old woman who presented with left-sided abdominal pain after colonoscopy with finding of splenic injury on CT scan. She was managed conservatively. We discuss the diagnostic and therapeutic approach to colonoscopic splenic injury.

## 1. Introduction

Approximately 14 million colonoscopies are performed in the US every year [1]. It is a safe procedure, with the main complications being bleeding (1-2%) and colonic perforation (0.1-0.2%) [2]. A rare and potentially fatal complication is splenic injury due to manipulation of the colonoscope through the large bowel.

When a patient presents with abdominal pain following colonoscopy, the main concern is that of colonic perforation [3]. CT scan of the abdomen and pelvis is highly sensitive for diagnosing colonic perforation [3]. Splenic injury is usually an unexpected finding of the test. Treatment is based on the hemodynamic status of the patient and on the degree of splenic injury seen on the CT scan [4].

## 2. Case Presentation

A 73-year-old woman underwent a screening colonoscopy that was initially reported as uneventful. Her medical history included atrial fibrillation treated successfully with radioablation. She was not on anticoagulation at the time of her exam. In her late 40s she underwent a total hysterectomy. Her medications included aspirin 81 mg daily.

Colonoscopy was done under moderate sedation with 75 mcg of fentanyl and 5 mg of midazolam given intravenously. The colonoscope was advanced to the cecum without difficulty. The exam was normal except for pandiverticulosis. The nurses' notes indicated that the patient had mild left-sided abdominal pain shortly after the procedure. This improved after passing gas and she was considered suitable for discharge.

**Figure 1:**
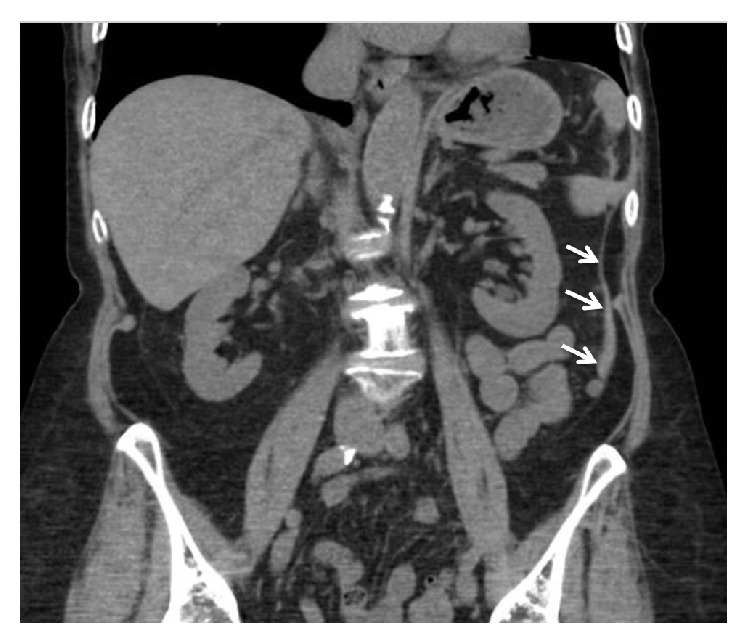
CT abdomen and pelvis without contrast. Coronal view showing small amount of high density perisplenic fluid (white arrows) tracking in the left paracolic gutter and extending into the pelvis.

Five hours after the procedure she called the endoscopist reporting increasing left-sided abdominal pain. There was no associated nausea, vomiting, fever, chills, dyspnea, or left shoulder pain. She was reevaluated in the endoscopist's office. On exam her vital signs were normal and there was moderate left-sided abdominal tenderness. There was no rebound or guarding. Concern for colonic perforation prompted a CT scan of the abdomen and pelvis, done without intravenous contrast. The study revealed high density perisplenic fluid consistent with blood, tracking in the left paracolic gutter (Figure 1), with no evidence of parenchymal damage. These findings indicated a grade 1 splenic injury (Table 1) and the patient was admitted for observation. She was managed conservatively with intravenous fluids. A modest overnight drop in hematocrit from 41% to 35% was noted, presumed to be partially related to hemodilution caused by the IV fluids. The next morning, the patient remained hemodynamically stable and her abdominal tenderness was much improved. She was discharged and seen in the office the following day with no further complaints of abdominal pain and no tenderness upon abdominal exam.

## 3. Discussion

Splenic injury is a rare complication of colonoscopy and is usually not a diagnostic consideration in patients who present with abdominal pain after the procedure. Since the first case report by Wherry et al., in 1974, around 100 cases have been reported [5–7]. The incidence has been estimated to be in the range of 0.00005% to 0.017% [8]. A review of ICD-9 coding for the combination of colonoscopy and splenic injury showed 437 cases out of 2,654,456 colonoscopies performed between the years 2000 and 2007, yielding a rate of 0.016% or about 1 out of 6000 [8]. The true incidence is likely higher due to underreporting resulting from a reluctance to publish information on procedural complications and lack of clear definition of colonoscopic splenic injury [7, 8]. Furthermore, mild cases may resolve before a diagnosis is made or may be misdiagnosed as nonspecific postcolonoscopy discomfort [7]. This complication has a female preponderance, with approximately 75% of cases being diagnosed in women [7, 9].

The mechanism of injury is presumably due to downwards traction on the splenocolic ligament or on adhesions around the spleen during colonoscopic manipulation causing a tear in the splenic capsule [2, 6, 8–10]. Capsular tear or parenchymal injury may also result from direct trauma inflicted on the spleen as the colonoscope passes through the splenic flexure [2, 8–10]. While anticoagulation and antiplatelet therapy do not, per se, cause splenic injury, they are likely to increase the degree of bleeding to a level that will be symptomatic enough to lead to a diagnosis [7]. The presence of splenomegaly has not been described as a contributing factor, but we may assume that an enlarged spleen will be more prone to injury, as reported in the trauma literature [11].

Patients with splenic injury during colonoscopy usually present within 24 to 48 hours, but presentation can be delayed for up to 10 days after the procedure [2, 7, 8, 10]. The most common complaint is left upper quadrant abdominal pain [8]. Additional symptoms include dizziness, palpitations, referred left shoulder pain from diaphragm irritation (Kehr's sign), and diffuse abdominal discomfort [8]. On physical examination patients may have localized left upper quadrant tenderness with or without signs of peritoneal irritation including guarding and rebound [12]. However, these signs and symptoms are nonspecific and the diagnosis of splenic injury is almost never made based on history and exam alone. The majority of cases are diagnosed by CT scan or at the time of laparotomy [7, 9, 13].

CT of the abdomen is the most reliable diagnostic tool for stable patients [4, 9, 13]. Findings suggestive of splenic injury include hemoperitoneum, hypodensity of the spleen, and contrast blush indicating extravasation of blood [13]. Although CT scan without intravenous contrast does not demonstrate parenchymal injury as well as a study with contrast, it is still quite sensitive. A retrospective analysis concluded that CT without contrast has a sensitivity of 93% in detecting splenic injury based on findings of a perisplenic hematoma or free abdominal fluid and strong clinical suspicion [13]. Furthermore, CT scan without contrast will quickly and accurately diagnose colonic perforation, which is the main concern when evaluating postcolonoscopy pain. The advantages of a study without contrast include the rapidity of the procedure and the ease of performing the test in an outpatient facility. It eliminates the need for IV access, checking creatinine, the risk of contrast induced nephropathy, and the concern for contrast allergy. We recommend, as injury is diagnosed on a CT scan without contrast, usually as an unexpected finding, not to repeat the test with contrast on a routine basis. In the few patients in whom more anatomic detail or evaluation of active bleeding is needed, the study can be repeated with contrast [13].

The American Association of Surgeons for Trauma (AAST) Splenic Injury Grading Scale is used to grade splenic injuries based on CT scan findings (Table 1) [4]. Treatment is based on the patient's clinical condition and the extent of splenic injury. There are three treatment options described in the literature: nonoperative management with fluid resuscitation and hemodynamic monitoring, interventional radiologic splenic artery embolization, and surgery [2, 6–8, 10]. Nonoperative treatment is the preferred choice, avoiding major surgery and preserving splenic immune function [12, 14]. Nonoperative management of splenic injury with fluid resuscitation, bed rest, and hemodynamic monitoring is more likely to succeed in patients with AAST grade I or II injury who are hemodynamically stable, lack peritoneal findings on exam, are younger than 55 years of age, and require little to no transfusion [12, 15, 16].

An estimated 85% of patients with blunt splenic injury in the past decade were managed nonoperatively [15]. In patients who fail this initial management and have ongoing bleeding but are hemodynamically stable, splenic artery embolization, which is highly successful in patients in blunt trauma, is the next step [16, 17]. The procedure has decreased the failure rate of nonoperative management from 12-13% to 2-3% [15]. The procedure appears to be safe, both in the elderly population with comorbidities and in patients who take medication that increases the risk of bleeding [15].

Surgical management is indicated in patients who fail conservative treatment and splenic artery embolization. The standard approach is open exploration. Laparoscopic surgery, which may be technically more demanding and more time consuming, is reserved for select patients with minimal hemorrhage and who are hemodynamically stable [18, 19]. Attempts at splenic salvage include splenorrhaphy and partial splenectomy. Splenectomy is indicated when all other management procedures have failed. Mortality from spleen injury is reported to be 5%, with a worse prognosis in patients who are diagnosed later [12].

## 4. Conclusion

In patients presenting with abdominal pain following colonoscopy, perforation is the primary concern. CT without contrast is recommended as the first diagnostic test. Splenic injury is so rare that it is almost never considered until diagnosis by CT imaging or laparotomy. This test, done without contrast, is sufficient to rule out colonic perforation and for evaluation of most splenic injuries. The scan can be repeated with IV contrast if necessary to obtain more anatomic information or to document active bleeding. Management is initially nonsurgical, which is successful in most patients. If it fails and the patient is hemodynamically stable, splenic artery embolization is the next step. Surgery is indicated, usually by open technique, when the patient is unstable or if bleeding continues after embolization attempt.

## Figures and Tables

**Table 1:** The American Association of Surgeons for Trauma (AAST). Splenic Injury Grading Scale (2008).

Grade	Injury	Criteria
I	Hematoma	Subcapsular, <10% of surface area
	Laceration	Capsular tear, <1 cm parenchymal depth

II	Hematoma	Subcapsular, 10–50% of surface area; intraparenchymal, <5 cm diameter
	Laceration	Subcapsular, 1 cm to 3 cm parenchymal depth that does not involve a trabecular vessel

III	Hematoma	Subcapsular, >50% of surface area or expanding; ruptured subcapsular or parenchymal hematoma; intraparenchymal hematoma, ≥5 cm diameter or expanding
	Laceration	>3 cm parenchymal depth or involving trabecular vessels

IV	Laceration	Laceration involving segmental or hilar vessels producing major devascularization of >25% of the spleen

V	Hematoma	Completely shattered spleen
	Laceration	Hilar vascular injury that devascularizes the spleen

^*∗*^Advance one grade for multiple injuries, up to grade III.

Reprinted from [4], with permission from Elsevier.

License number 3917700781230.

Source: http://www.sciencedirect.com/science/article/pii/S1072751508011010.
